# Supplementary material for: Al2O3-Based Hollow Fiber Membranes Functionalized by Nitrogen-Doped Titanium Dioxide for Photocatalytic Degradation of Ammonia Gas
Source: Membranes (Basel). 2022 Jul 6;12(7):693. doi: 10.3390/membranes12070693 (PMC9320378; doi:10.3390/membranes12070693)
Supplement: Supplementary file 1 [file membranes-12-00693-s001.zip › membranes-1769516-supplementary.pdf]

Supplementary Material

Al_2O_3-based hollow fiber membranes functionalized by nitrogen-doped titanium dioxide for photocatalytic degradation of ammonia gas

Edoardo Magnone[1], Jae Yeon Hwang[1], Min Chang Shin[1], Xue Long Zhuang[1], Jung In Lee[1] and Jung Hoon Park[1,*]

[1] Department of Chemical and Biochemical Engineering, Dongguk University, 30, Pildong-ro 1 gil, Jung-gu, Seoul, 04620, South Korea

* Correspondence: author. Tel: +82-2-2260-8598. Fax: +82-2-2260-8729. E-mail address: pjhoon@dongguk.edu (J.H.Park).

Table S1. Chemicals used in the phase inversion process to prepare the Al_2O_3 hollow fiber membrane and the dip-coating deposition of TiO_2 films.

Material	Provider	Note
α-Al_2O_3	Kceracell (Korea)	Particle size= 0.5 μm
Polyethersulfone (PESf)	Ultrason® (DEU)	-
Polyvinylpyrrolidone (PVP)	Sigma Aldrich (USA)	99.5%
Polyvinyl alcohol (PVA)	Sigma Aldrich (USA)	MW=1800Da
Titanium (IV) isopropoxide (TTIP)	Sigma Aldrich (USA)	97%
Tetraethyl orthosilicate (TEOS)	Sigma Aldrich (USA)	98%
1-methyl-2-pyrrolidinone anhydrous	Samchun Pure Chemical Co. Ltd (Korea)	99.5%
Urea	Samchun Pure Chemical Co. Ltd (Korea)	99%

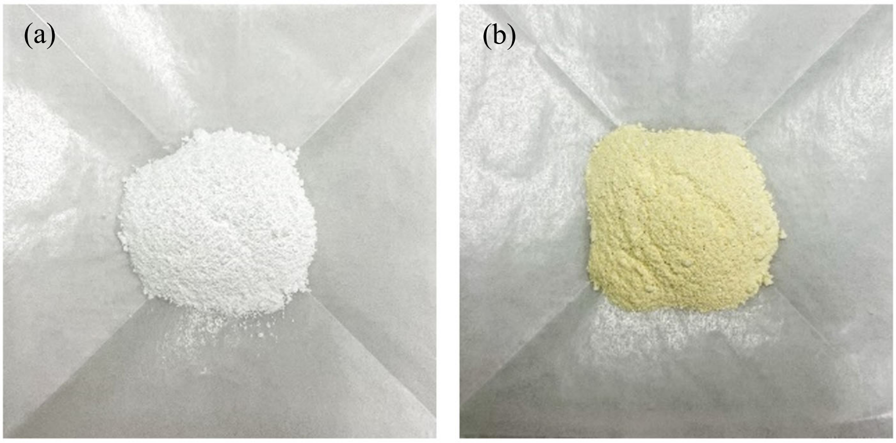

Figure S1. Digital photographs of the prepared (a) undoped TiO_2 and (b) N-TiO_2 photocatalysts.

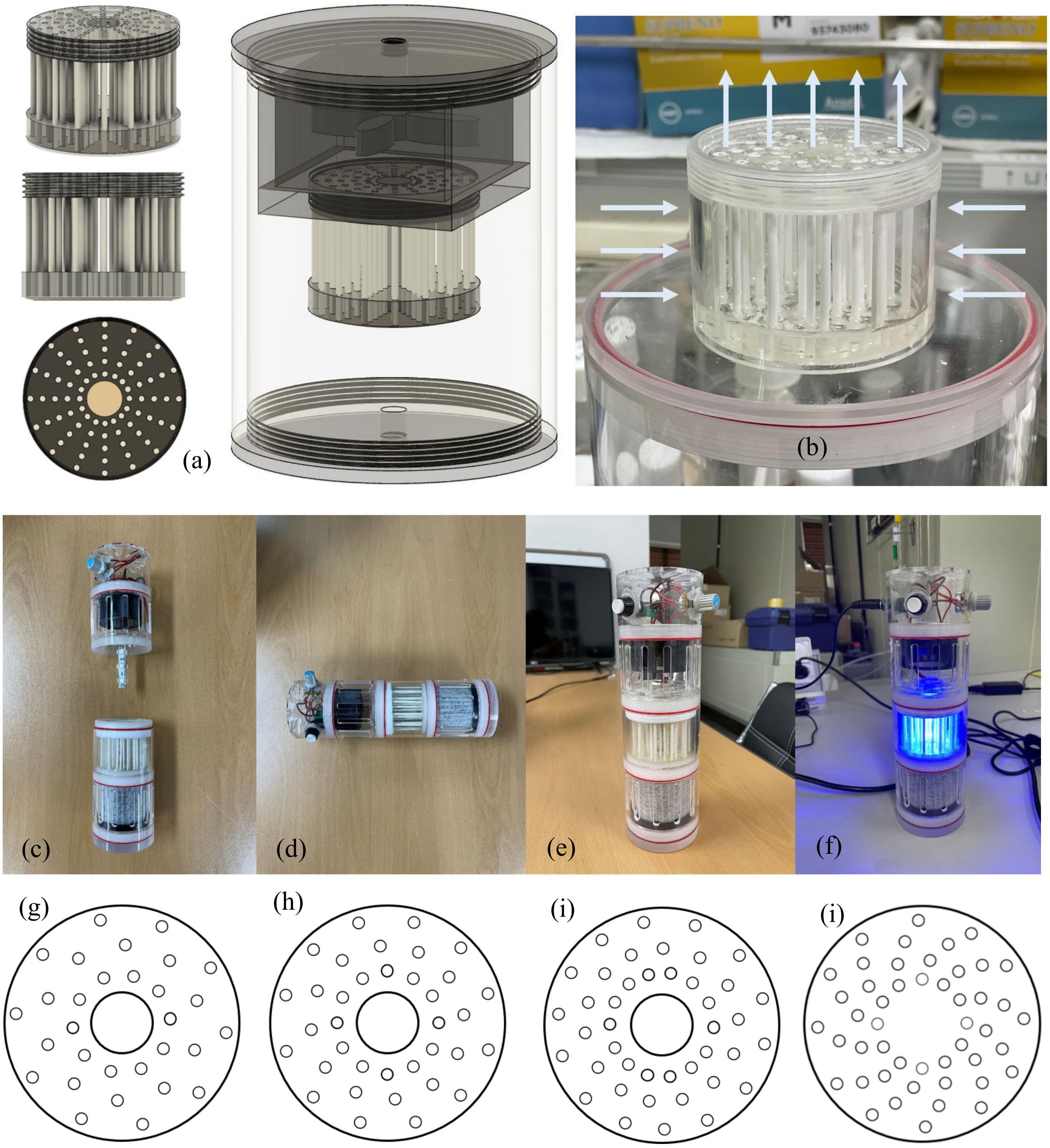

Figure S2. (a) Photocatalytic membrane reactor (below) with the brushless DC fan (above), (b) NH_3 flux through the photocatalytic membrane reactor, (c) disassembled photocatalytic membrane reactor, assembled photocatalytic membrane reactor, (e) without light photocatalytic membrane reactor, (f) with light photocatalytic membrane reactor, and photocatalytic membrane reactor based on (g) 30, h) 36, (i) 42, and (l) 48 functionalized Al_2O_3-based hollow fiber membranes.

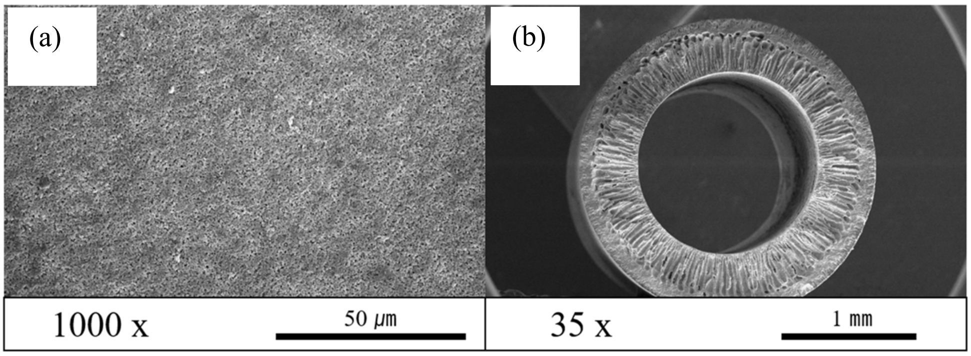

Figure S3. SEM images of (a) the surface and (b) a cross section of an Al_2O_3 hollow fiber membrane after a high-temperature sintering process (1300°C; 3 hours).

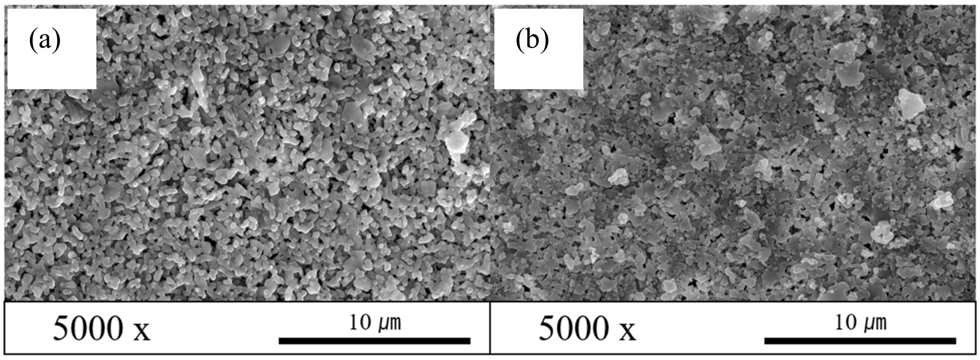

Figure S4. SEM images of the (a) Al_2O_3-based hollow fiber membrane surface and (b) Al_2O_3-based hollow fiber membrane surface functionalized by N-TiO_2 photocatalysts.

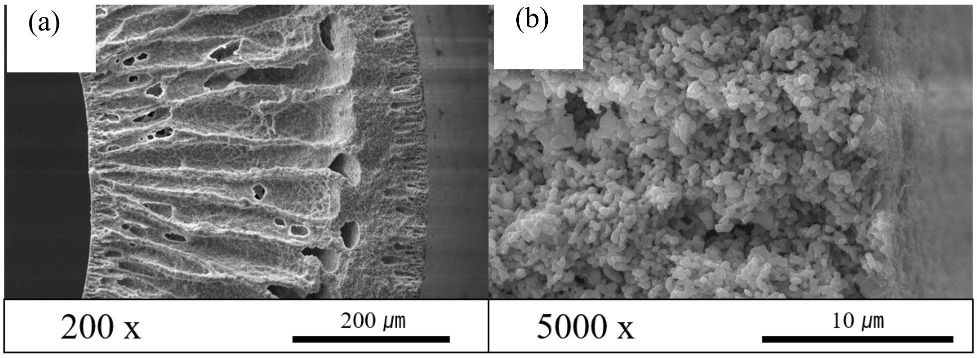

Figure S5. SEM images of the cross section of an Al_2O_3-based hollow fiber membrane surface functionalized by N-TiO_2 photocatalysts with two different a magnifications: (a) 200 μm and (b) 10 μm.

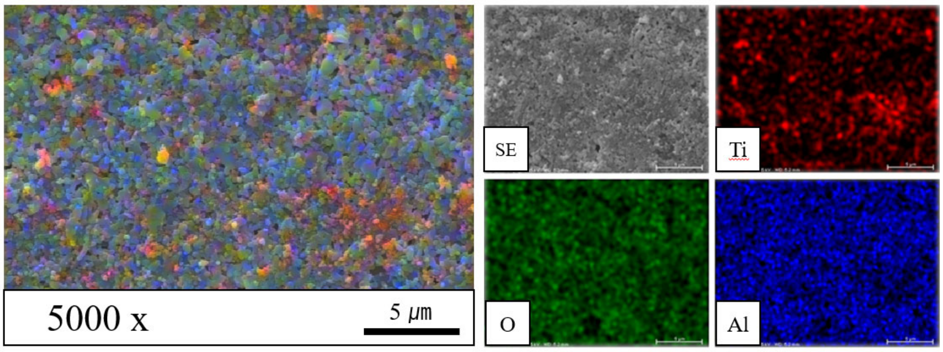

Figure S6. EDS mapping images of the surface of the Al_2O_3-based hollow fiber membranes functionalized by N-TiO_2 photocatalysts.

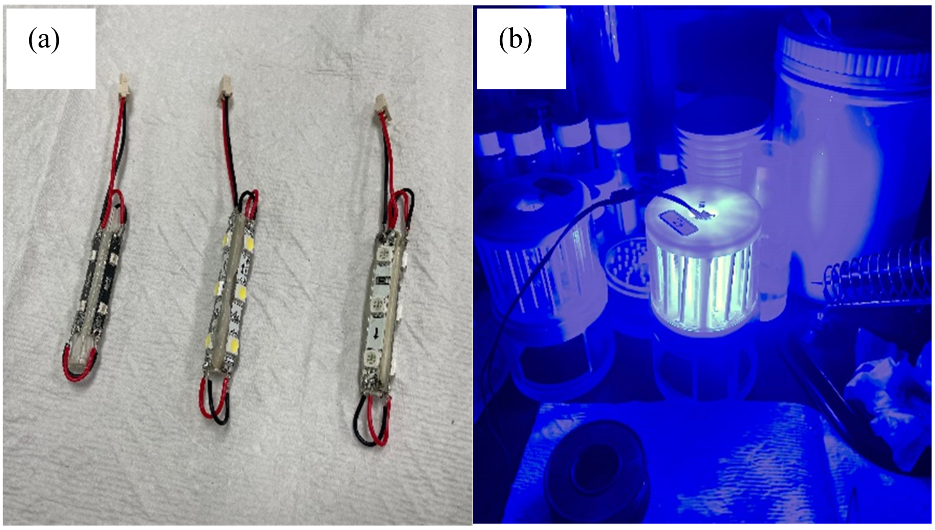

Figure S7. Digital photographs of the (a) LED lamps (white, blue, and ultraviolet) in the triangular shape, and (b) an LED lamp mounted in the middle of the photocatalytic membrane reactor.

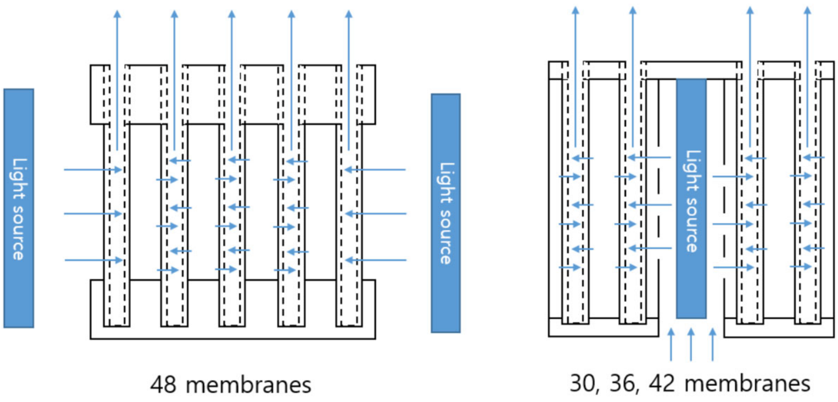

Figure S8. Prototype photocatalytic membrane reactors with (a) external and (b) internal light sources.

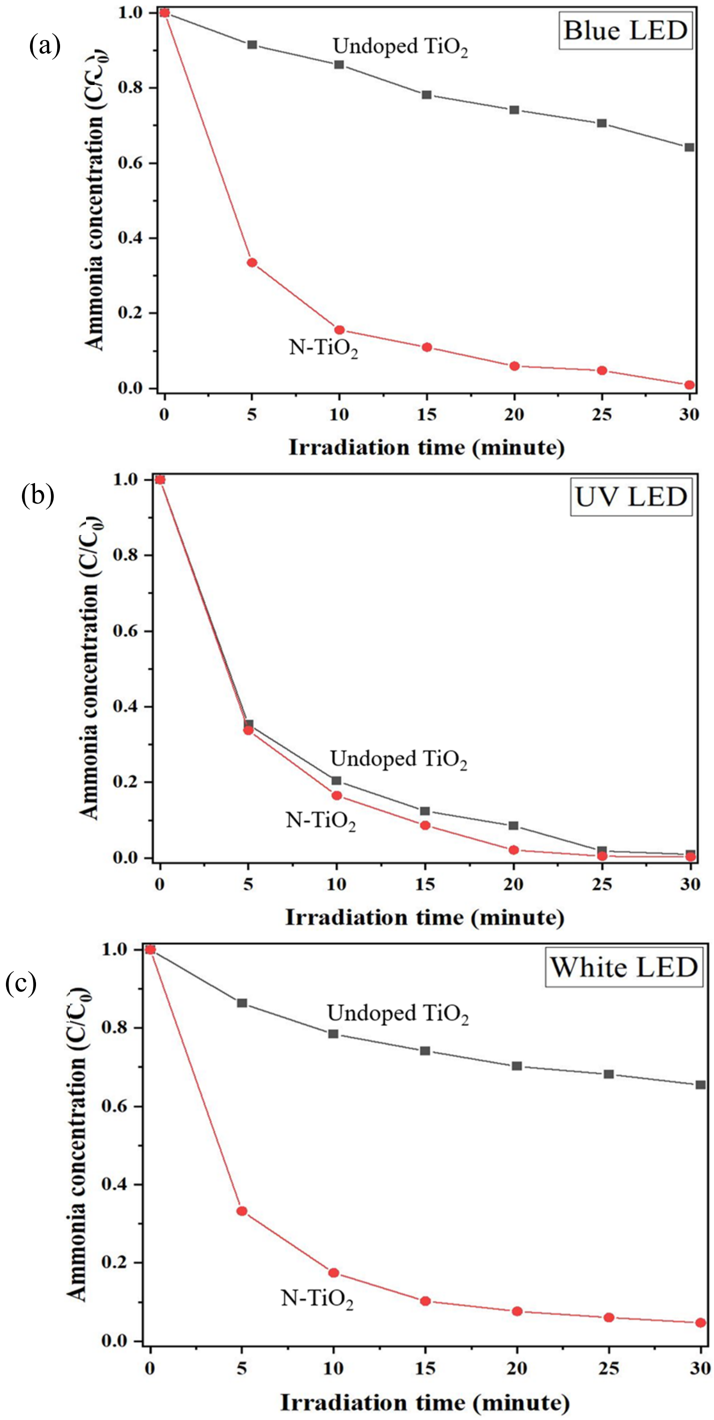

Figure S9. NH_3 heterogeneous photocatalytic membrane reactor capacity of a 36 Al_2O_3-based hollow fiber membranes functionalized by undoped TiO_2 and N-TiO_2 photocatalysts under LED light sources: (a) white, (b) blue, and (c) ultraviolet.
